# Advantages of AI-based whole blood film scanning for blast detection in markedly leucopenic blood films

**DOI:** 10.1007/s00277-025-06473-0

**Published:** 2025-07-15

**Authors:** Geng Wang, Lin Zheng, Zhejun Fang, Guoju Luo, Qian Chen, Qi Qi Zhang, Bo Shen Wu, Xin Wang, Rongrong Cheng, Ping Deng, Binyao Zhang, Jing Jin, Wei Wu

**Affiliations:** 1 Department of Clinical Laboratory, Peking Union Medical College Hospital, Beijing, 100730 China; 2 Beijing Xiaoying Technology Co., Ltd, Beijing, 100085 China

**Keywords:** Artificial intelligence, Whole-slide scanning, Cygnus instrument, Leucopenic, Blast cells

## Abstract

To evaluate performance of AI-assisted blast detection via whole blood film scanning in cases of severe leukopenia. From August to October 2023, we collected 157 patient specimens with white blood cell (WBC) counts of ≤2.0 × 10⁹/L. We then evaluated and compared the blast detection capabilities of an artificial intelligence-based system (Cygnus instrument) against the CellaVision DI-60 for WBC analysis. Additionally, this study aimed to assess the comparative performance of these two automated blood cell morphology analyzers based on their underlying operational principles. Following manual microscopy confirmation, 17 cases showing ≥ 1 blast cell in peripheral blood smear were identified among 157 specimens with WBC ≤ 2.0 × 10⁹/L. The Cygnus whole-slide scanning mode detected all 17 cases (sensitivity: 100%), whereas the Cygnus 200-cell mode detected only 9 cases (9/17), and the CellaVision DI-60 200-cell mode detected 8 cases (8/17). In leukopenic patients (WBC ≤2.0×10⁹/L), utilizing Cygnus' whole-slide scanning capability substantially improves blast identification rates, potentially enabling earlier detection of hematologic abnormalities.

**Supplementary Information:**

The online version contains supplementary material available at 10.1007/s00277-025-06473-0.

## Introduction

Blast cells are hematopoietic progenitor cells in the bone marrow that differentiate into mature blood cell lineages. In accordance with standardized hematological reporting protocols, comprehensive evaluation of blast cells - even at minimal concentrations - is imperative in peripheral blood smear analysis. Particular attention must be paid to identifying morphological aberrations indicative of: ①Malignant features; ②Pathognomonic nuclear abnormalities; ③Aberrant cytoplasmic characteristics. All such findings require detailed documentation in diagnostic reports and should be designated as “critical morphological findings”, necessitating urgent clinical notification and intervention.

The International Council for Standardiszation in Haematology (ICSH) recommends the use of AI algorithms for pre-classifying cells and employing digital imaging (DI) technology in blood film analysis, setting a forward-looking standard for the industry [1]. Although fulaly automated hematology analyzers substantially enhance laboratory testing efficiency and standardization, they exhibit notable limitations in cellular morphological assessment. These systems frequently fail to accurately characterize intracellular morphological features and subtle structural abnormalities, potentially leading to diagnostic inaccuracies and false-negative results that compromise clinical decision-making [2]. In contrast, manual peripheral blood smear microscopy remains indispensable for comprehensive disease diagnosis and prognosis, as it enables direct visualization of pathognomonic cellular alterations that may indicate underlying pathology [3].

Current hematological practice guidelines emphasize the critical importance of meticulous morphological evaluation, particularly in leukopenic patients (WBC < 2.0 × 10⁹/L) who demonstrate increased infection risk and potential for life-threatening complications [4]. This examination becomes especially crucial when leukocyte density falls below 10 cells per microscopic field (400× magnification), necessitating systematic review of multiple fields to ensure diagnostic accuracy. Special attention must be given to the identification and characterization of blast cells, whose presence may signify clinically significant hematological disorders.

The diagnostic accuracy of peripheral blood smear microscopy is inherently limited by inter-observer variability stemming from differences in examiner expertise and experience [4, 5]. This fundamental limitation has driven the rapid development of automated digital morphology analysis systems designed to standardize blood smear interpretation. To objectively evaluate the performance of these emerging technologies, we conducted a comprehensive comparative study of two automated hematological morphology analyzers employing distinct analytical principles in our clinical laboratory setting.

The study was designed to:

1. Assess the diagnostic reliability of each platform.
2. Systematically compare their respective analytical strengths and limitations.
3. Provide evidence-based recommendations for clinical implementation.

The study aims to enhance laboratory testing standardization, improve diagnostic accuracy, and optimize workflow efficiency through rigorous methodological comparison.

## Materials and methods

### Data acquisition

From August to October 2023, we prospectively collected 157 fresh EDTA-K2 anticoagulated venous blood specimens meeting strict inclusion criteria (WBC ≤ 2.0 × 10⁹/L) at our tertiary medical center. All samples were maintained under standardized conditions (ambient temperature: 18–22 °C) and processed within 2 h of collection to ensure optimal cellular preservation and minimize preanalytical variability.

### Instrumentation

The following automated systems and equipment were employed in this study:

1. Cygnus Automated Blood Cell Morphology Analysis System (Beijing Xiaoying Technology Co., Ltd., China).
2. CellaVision DI-60 Automated Digital Cell Morphology System (CellaVision AB, Lund, Sweden).
3. Sysmex SP-10 Automated Slide Maker and Stainer (Sysmex Corporation, Kobe, Japan).
4. Olympus CX33 Clinical Microscope (Olympus Corporation, Tokyo, Japan).

### Comparative analysis of instrument methodologies

#### Whole slide imaging versus selective cell counting

The Cygnus hematological analysis system integrates state-of-the-art whole-slide imaging (WSI) technology featuring real-time dynamic focusing functionality, which facilitates comprehensive digitization of complete peripheral blood smears. This technological innovation provides three significant diagnostic advantages:

1. Substantially improved imaging efficiency through high-speed acquisition of high-resolution (≥ 0.09 μm/pixel) cellular images.
2. Enhanced objectivity in cellular morphological evaluation, particularly crucial for cases of severe leukopenia (defined as WBC count < 1.0 × 10⁹/L).
3. Complete enumeration capability for all nucleated cells present on the entire slide surface.

Notably, the full-slide scanning modality exhibits superior sensitivity for the detection of blast cells compared to conventional selective counting methods (e.g., the 200-cell analysis mode in both the Cygnus and CellaVision DI-60 systems), thereby significantly reducing false-negative rates in clinical diagnostics.

#### Adaptive versus fixed scanning area protocols

Both analyzers offer selective leukocyte enumeration modes (200-cell count), with primary sampling focused on the monolayer-body/tail junction region. However, the Cygnus system features a unique adaptive scanning algorithm that:

- Dynamically expands the examination area to achieve target cell counts in leukopenic samples.
- Maintains standardized counting precision regardless of cellularity.
- Provides operator-adjustable scanning parameters for optimized workflow.

In contrast, the CellaVision DI-60 operates with a fixed scanning area, which may compromise counting accuracy in specimens with extremely low cellularity (< 5 cells/µL). This technical limitation becomes particularly evident when analyzing bone marrow-suppressed patients or minimal residual disease cases.

#### Vision transformer-based deep learning versus ANN-based machine learning

The CellaVision DI-60 system utilizes an artificial neural network (ANN)-based machine learning approach [6] that depends on manually engineered feature extraction and conventional pattern recognition methodologies. In contrast, the Cygnus system implements a sophisticated Vision Transformer deep learning architecture [7] that leverages self-attention mechanisms and autonomous hierarchical feature learning, enabling comprehensive end-to-end image analysis and interpretation.

### Experimental methods

Peripheral blood samples were initially processed using the Sysmex XN-9000 hematology analyzer. In accordance with standardized hematological reporting guidelines, specimens demonstrating leukopenia (WBC ≤ 2.0 × 10⁹/L) were selected for further analysis. Each qualifying sample underwent parallel evaluation using four distinct methodologies:

1. CellaVision DI-60 automated morphology system (200-cell analysis mode).
2. Cygnus automated morphology system in three scanning configurations:

- 200-cell analysis mode.
- Whole-slide scanning mode.

All automated analyses were subjected to rigorous morphological validation by our laboratory’s hematopathology team. The validation protocol consisted of:

- Primary evaluation by a senior cytomorphology specialist.
- Secondary review by an independent expert.
- Adjudication of discordant results by a third specialist (board-certified hematopathologist).

Finalized morphological classifications were archived in a secure database for subsequent statistical evaluation.

### Statistical analysis

In this study, statistical analysis was performed using the independent-samples T Test to compare means between two independent groups. This test was chosen because it is appropriate for comparing the means of two independent samples when the data are normally distributed and have homogeneous variances. A cut-off value of *p* < 0.05 was considered statistically significant.

The statistical analysis was conducted using IBM SPSS Statistics, version 28.0 (IBM Corporation, Armonk, NY, USA).

## Results

### Cases of leukopenia with blast cells

Among 157 tested samples with leukopenia (WBC ≤ 2.0 × 10⁹/L), morphological review by hematology experts confirmed the presence of blast cells in 17 cases (10.8%). As shown in Table S1, these 17 patients exhibited the following characteristics:

- **Department distribution**: Patients were not exclusively admitted to hematology or oncology departments but presented across multiple clinical specialties.
- **Initial diagnoses**: The primary admitting diagnoses were not uniformly hematologic disorders.
- **Clinical manifestations**: Some patients initially presented with nonspecific symptoms, such as fever, cough, or oral ulcers.

These findings suggest that in clinical practice, even in cases of marked leukopenia without obvious hematologic indications, peripheral blood smear review for blast cells remains crucial for early detection of potential underlying malignancies.

### Comparative detection rates of blast-positive samples across analytical platforms

**Fig. 1:**
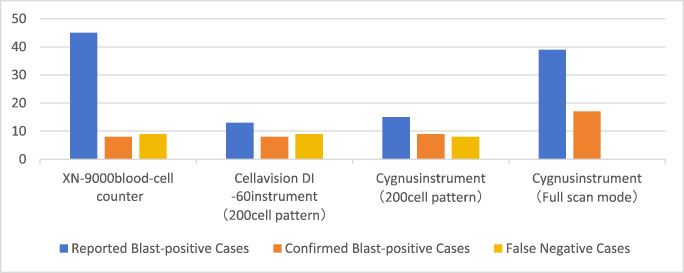
Comparative performance analysis of blast cell detection

Among the 157 clinical specimens evaluated, significant variability in blast cell detection accuracy was observed between instruments (Table S2, Fig. 1). Both false-positive (erroneous blast flagging) and false-negative (missed blast cell) events were documented, revealing platform-dependent limitations in automated hematological analysis. The discordant results suggest fundamental differences in either analytical sensitivity or morphological recognition algorithms among the tested systems.

Our evaluation of blast cell detection accuracy revealed significant discrepancies in the XN-9000 hematology analyzer’s performance. While the instrument generated blast flags in 45 cases, subsequent manual microscopy confirmation (Table S2) identified only 8 true-positive instances (positive predictive value: 17.8%). More critically, we observed 9 false-negative cases where blast cells were microscopically evident but undetected by the XN-9000. These findings suggest systemic limitations in the instrument’s blast detection algorithm, particularly concerning both specificity and sensitivity.

In the 200-white blood cell analysis mode, comparative evaluation revealed differential detection capabilities between the automated systems. The Cygnus platform identified 9 blast-positive specimens compared to 8 detected by the CellaVision DI-60 system. Detailed discordance analysis demonstrated that Cygnus detected 3 blast-containing samples missed by DI-60 (false negatives for DI-60), while DI-60 identified 2 cases undetected by Cygnus (false negatives for Cygnus). This reciprocal detection failure pattern, observed despite both instruments scanning the body-tail junction of blood smears, suggests fundamental differences in their scanning algorithms at this critical morphological region. The partial non-overlap in detection (Jaccard similarity index = 0.67) implies complementary rather than redundant diagnostic value when used in tandem.

The Cygnus platform achieved 100% blast detection rate in whole-slide scanning mode, demonstrating superior performance compared to both the CellaVision DI-60 system and its own 200-white blood cell (WBC) analysis mode. This enhanced detection capability stems from Cygnus’ comprehensive whole-slide scanning approach, which minimizes the probability of missing blast cells that might be overlooked in limited-field analysis. These findings underscore the inherent limitations of regional scanning methodologies in blast cell identification.

All instruments and their respective scanning modes exhibited false-positive identifications (Fig. 3). This phenomenon primarily results from intentionally heightened sensitivity settings designed to prevent blast cell omission. Morphological similarities between blast cells and other cell types - including abnormal lymphocytes and promyelocytes - in terms of cell size, chromatin pattern, and cytoplasmic granularity contributed to these misclassifications.

**Fig. 2:**
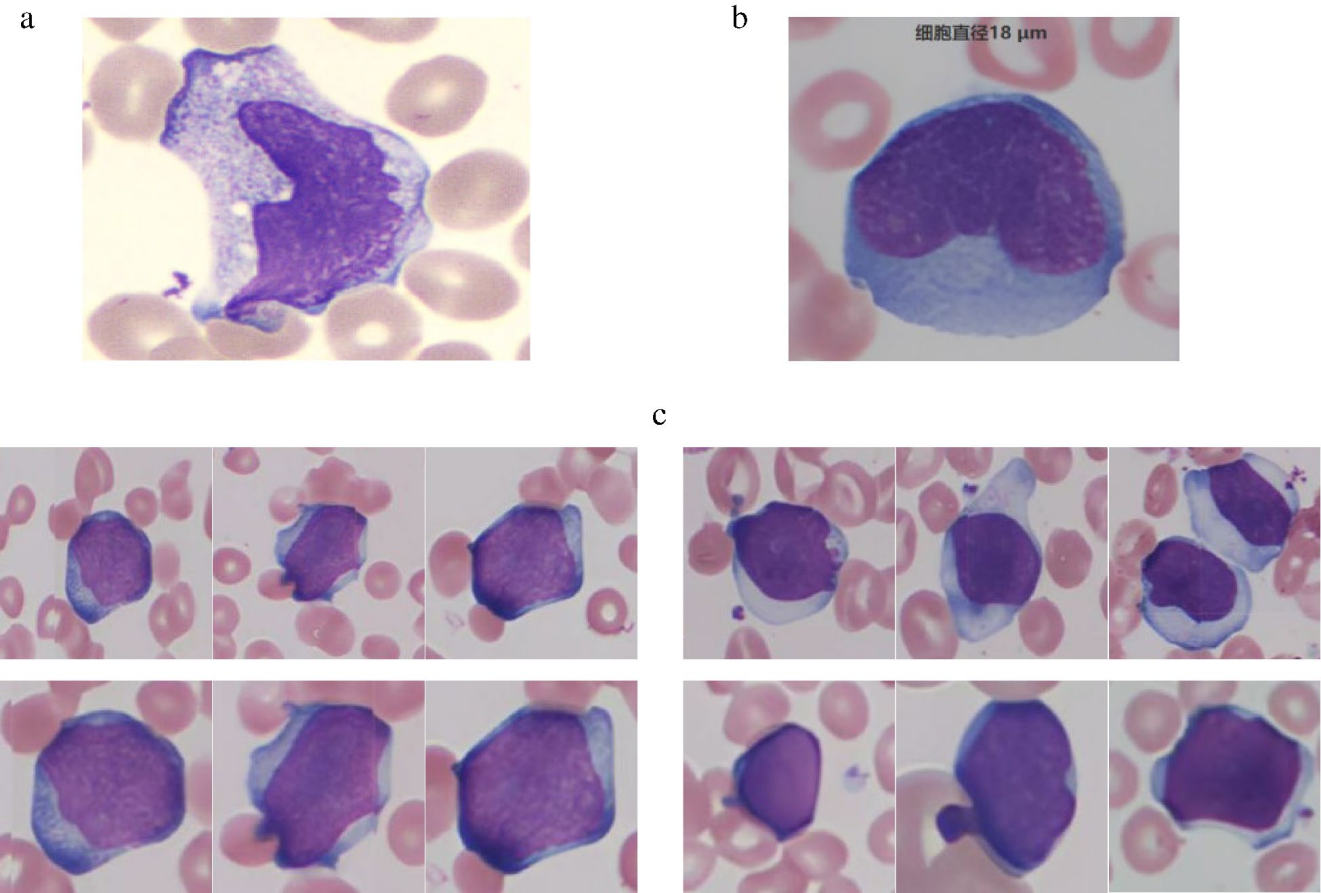
**a**: Blast cell misidentification by CellaVision DI-60 system(Actual cell type:Monocyte). **b**: Blast cell misidentification by Cygnus platform(Actual cell type:Reactive lymphocytes). **c**: Blast cells identification by Cygnus platform

### Comparative analysis of instrument performance

**Fig. 3:**
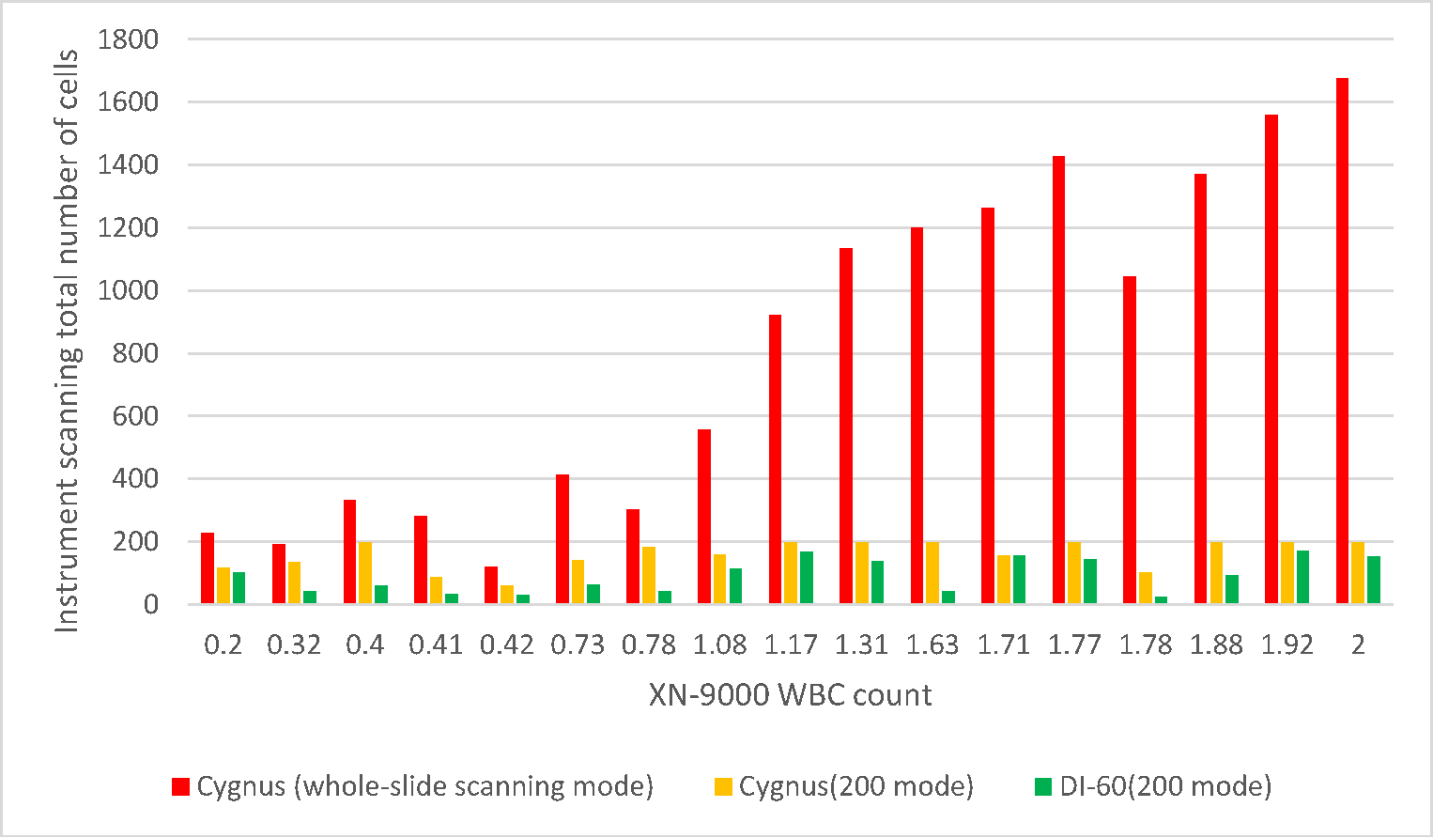
Results of various instruments and instrument modes

In this section, we conducted a detailed analysis of the scanning results from 17 samples (Table S3, Fig. 3) to investigate potential causes of detection discrepancies, with particular focus on scanning range and total white blood cell (WBC) count.

#### Association between scanned WBC count and blast cell detection rate

Our analysis revealed a positive correlation between the total number of scanned WBCs and blast cell detection probability. The Cygnus platform demonstrated significantly higher total WBC counts in both whole-slide scanning mode and 200-cell analysis mode compared to the CellaVision DI-60 system (*p* < 0.05).

During evaluation of 157 leukopenic samples (WBC ≤ 2.0 × 10⁹/L), we observed distinct scanning performance characteristics: The Cygnus system achieved the target 200-cell count in 61.1% of samples when operating in 200-cell analysis mode. In contrast, the CellaVision DI-60 system failed to reach 200 cells in the majority of samples under identical analysis conditions. This discrepancy in cellular acquisition efficiency may contribute to the observed differences in blast cell detection sensitivity between platforms.

The observed discrepancies in leukocyte enumeration between instruments can be attributed to two primary factors:

## Leukopenic sample limitations

In cases of severe leukopenia (WBC < 1.0 × 10⁹/L), both instruments occasionally failed to achieve the target 200-cell count despite exhaustive scanning of the body-tail junction region. This technical limitation stems from the inherently low cellularity in such specimens, potentially compromising statistical reliability in morphological analysis.

## Non-leukocyte misclassification

The DI-60 system demonstrated a notable tendency for cellular misidentification, frequently classifying non-leukocytic elements (including: Cellular debris, Smear artifacts, Platelet aggregates, Nucleated erythrocytes) as legitimate white blood cells. This systematic error results in artificially inflated cell count readings, suggesting that the actual analyzable leukocyte population may be lower than instrument-reported values (see Fig. 3 for exemplar misclassification cases).

These findings underscore the importance of manual verification in leukopenic samples, particularly when clinical decisions depend on precise cellular enumeration.

### Impact of scanning region selection on blast cell detection

When operating in 200-cell analysis mode, both analyzers exhibited complementary detection failures, with each instrument missing blast cells that were identified by the other. This reciprocal detection pattern persisted despite standardized scanning at the body-tail junction, suggesting fundamental differences in the instruments’ scanning algorithms at this critical morphological region.

The Cygnus platform demonstrated significantly enhanced blast detection sensitivity in whole-slide scanning mode compared to both its own 200-cell analysis mode and the CellaVision DI-60 system (*p* < 0.01). This performance advantage stems from Cygnus’ comprehensive slide coverage, which eliminates the sampling bias inherent in restricted-field analysis. The observed improvement in detection rate underscores the limitations of region-specific scanning methodologies.

**Fig. 4:**
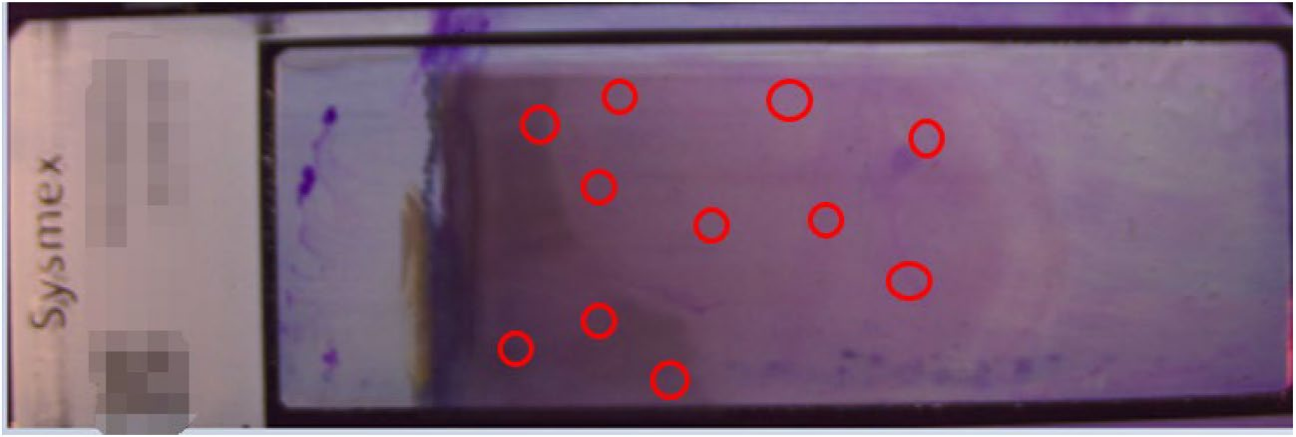
Spatial distribution of blast cells on blood smear slides (Circles indicate the locations of blast cells)

Morphometric analysis revealed that only 32% of detected blast cells were located at the conventional body-tail junction (Fig. 4). The majority (68%) were distributed throughout the slide’s thick-end region, forming distinct cellular clusters. This non-uniform distribution pattern explains the suboptimal performance of region-limited scanning protocols, particularly in leukopenic samples (WBC < 1.0 × 10⁹/L).

## Discussion

### Clinical significance of blast cell detection

Current guidelines for standardized hematological reporting emphasize that even rare blast cells observed in peripheral blood smears require detailed morphological evaluation, including assessment for malignant features, nuclear atypia, and cytoplasmic abnormalities. These findings must be documented in the clinical report and flagged as critical values, necessitating immediate therapeutic intervention. While manual microscopic review remains the gold standard, comprehensive screening of all leukocytes in leukopenic samples (WBC ≤ 2.0 × 10⁹/L) is prohibitively time-intensive. Automated whole-slide scanning systems address this diagnostic challenge by enabling rapid, large-scale blast cell screening with high sensitivity.

### Diagnostic trajectories of blast-positive cases

Summary of clinical data from 17 patients revealed that individuals with blast cells detected in peripheral blood smears were not exclusively admitted to hematology or oncology departments, nor were their initial diagnoses uniformly hematologic disorders. Some patients sought medical attention for symptoms such as fever, cough, or oral ulcers. If laboratory personnel fail to recognize blast cells during routine examination, clinicians may overlook critical diagnostic clues, potentially delaying optimal intervention. Therefore, for patients presenting with leukopenia during initial evaluation, we recommend performing comprehensive peripheral blood smear scanning using Cygnus automated microscopy system to ensure sensitive detection of blast cells.

### Optimization of diagnostic protocols

Comparative performance data strongly support adopting the Cygnus whole-slide scanning protocol as first-line screening for leukopenic patients, based on:

- **Superior detection sensitivity** (100% vs. 52.9% in 200-cell mode; *p* < 0.001).
- **Reduced spatial sampling bias** (68% of blasts located outside standard body-tail junction).
- **Operational efficiency** (mean scan time: 10 min vs. 40 min for manual review).

## Conclusion

When WBC ≤ 2.0 × 10^9/L, Cygnus instrument whole-slide scanning mode can be quickly scanned and the number of all white blood cells in the slide can be counted, which makes the white blood cells classification more statistically significant. The whole-slide scanning mode does not miss blasts at the thick and thin ends of the slide, resulting in more accurate results; With the function of cell positioning, the cells in doubt can quickly find their position in the blood film, saving time for microscopic examination.

Commercial AI-based hematology analyzers demonstrate strong performance in analyzing WBCs, RBCs, and PLTs, with high classification accuracy. However, they still face limitations in detecting rare cell types and blood-borne parasites [1]. The Cygnus instrument addresses one such limitation—identifying blasts in severe leukopenia cases—where current AI technologies fall short.

Moving forward, we aim to:

- Reduce scanning time in whole-slide imaging mode,
- Enhance blast cell recognition accuracy,
- Improve detection of blood-borne parasites, and.
- Expand capabilities to process a broader range of rare cell types.

## Related work

In 2022, Nam et al. [8] demonstrated that the CellaVision DI-60 faces limitations when analyzing nearly 200 leukocyte pre-classification samples, particularly in moderate leukopenia. The process becomes significantly prolonged in cases of severe leukopenia, often complicating the accurate enumeration of 200 leukocytes. In contrast, the Cygnus system, with its full-slide scanning capability, achieves higher leukocyte counts with greater efficiency.

Moreover, the Cygnus instrument employs deep learning algorithms, which outperform the conventional machine learning methods used by the CellaVision DI-60. This advanced computational approach enhances recognition accuracy, improves analytical stability, reduces subjective bias, and minimizes false inclusions of non-leukocytic elements, thereby ensuring superior result objectivity. These improvements highlight the Cygnus system’s potential to redefine leukocyte differential counting in clinical diagnostics.

Further supporting these findings, Yoon et al. [9] reported that the CellaVision DI-60 demonstrates suboptimal repeatability and intra-laboratory precision in blast cell detection. Specifically, the %CV (coefficient of variation) for blast cells exceeded 30% across normal, mild, moderate, and severe leukocyte counts. For context, %CV ≤ 10% reflects excellent repeatability, 10–20% indicates good precision, 20–30% is marginally acceptable, while %CV > 30% signifies unreliable performance.

Additionally, Zhao et al. [10] observed that the CellaVision DI-60 showed significant mean deviations from manual microscopy across all leukocyte subtypes, especially in cases of moderate-to-severe leukocytosis and leukopenia. Among four abnormal cell categories, the discrepancy was most pronounced for blast cells.

The underlying cause of this inaccuracy may stem from sampling bias, where an elevated WBC concentration per field coupled with a reduced counting area skews results. This issue can be alleviated by increasing the counted cells to 300–500, a strategy effectively implemented by the Cygnus system via full-slide scanning, proving particularly advantageous in leukopenic samples.

## Electronic supplementary material

Below is the link to the electronic supplementary material.

Supplementary Material 1

## Data Availability

The data are authentic and available in the manuscript.We do not need to obtain the patient’s informed consent, because we found that the white blood cells were below 3.0 during the routine specimen test, which met the rules of this experiment, and the microscopic examination of the push slide used two readers, and the advantages of the Cygnus instrument were found in the process.

## Declarations

### **Human and animal protection**

A total of 157 EDTA-K2 anticoagulated whole blood samples were collected from outpatients and inpatients at Peking Union Medical College Hospital between August and October 2023. This study was approved by the Institutional Review Board (IRB) of Peking Union Medical College Hospital (Approval No. S-T594) and conducted in compliance with the Declaration of Helsinki (1964). All samples were stored at room temperature (18–22 °C) and processed within 2 h of collection.

### Author’s statement

All authors agree to publication.
