# Supplementary material for: Advantages of AI-based whole blood film scanning for blast detection in markedly leucopenic blood films
Source: Ann Hematol. 2025 Jul 15;104(10):5415–22. doi: 10.1007/s00277-025-06473-0 (PMC12619778; doi:10.1007/s00277-025-06473-0)
Supplement: Supplementary file 1 — Supplementary Material 1 [file 277_2025_6473_MOESM1_ESM.docx]

**Table S1 Clinical Characteristics and Diagnostic Outcomes of 17 Patients with Blast-Positive Leukopenia**

| **Case No.** | **Initial Diagnosis/Chief Complaint** | **Final Diagnosis** | **Treating Department** |
| --- | --- | --- | --- |
| **1** | **Oral ulcers, fever** | **Acute myeloid leukemia-M2 type** | **Hematology department** |
| **2** | **Ovarian cancer** | **Ovarian cancer** | **Internal medicine** |
| **3** | **Skin bruising for 1 week** | **Acute leukemia** | **Hematology department** |
| **4** | **Fever and cough** | **Acute myeloid leukemia-M2 type** | **Hematology department** |
| **5** | **Pain in left back and leg** | **Undifferentiated small round cell sarcoma** | **Department of orthopedics** |
| **6** | **Acute myeloid leukemia** | **Acute myeloid leukemia** | **Internal medicine** |
| **7** | **Cervical carcinoma** | **Cervical carcinoma** | **Gynecological tumors** |
| **8** | **Hemoptysis, decreased blood triad** | **Chronic NK value-added disease** | **Hematology department** |
| **9** | **Myelodysplastic syndrome** | **Myelodysplastic syndrome** | **Internal medicine** |
| **10** | **Acute leukemia** | **Acute leukemia** | **Hematology department** |
| **11** | **Rhabdomyosarcoma of the vagina** | **Rhabdomyosarcoma of the vagina** | **Gynecological tumors** |
| **12** | **Cervical cancer, myelosuppression** | **Cervical cancer, myelosuppression** | **Radiotherapy department** |
| **13** | **Cervical carcinoma** | **Cervical carcinoma** | **Radiotherapy department** |
| **14** | **Ovarian cancer** | **Ovarian cancer** | **Gynecological tumors** |
| **15** | **Vaginal Bleeding** | **Gestational trophoblastic tumor** | **Gynecological tumors** |
| **16** | **Myelodysplastic syndrome** | **Myelodysplastic syndrome** | **Internal medicine** |
| **17** | **Pancreatic cancer** | **Pancreatic cancer** | **Internal medicine** |

**Table S2: Comparative Performance Analysis of Blast Cell Detection**

| **Instruments** | **Reported Blast-positive Cases** | **Confirmed Blast-positive Cases** | **False Negative Cases** |
| --- | --- | --- | --- |
| **XN-9000 blood cell analyzer** | **45** | **8** | **9** |
| **CellaVision DI-60 instrument (200 White Blood Cell mode)** | **13** | **8** | **9** |
| **Cygnus instrument (200 White Blood Cell mode)** | **15** | **9** | **8** |
| **Cygnus instrument (whole-slide scanning mode )** | **39** | **17** | **0** |

**Table S3: Results of Various Instruments and Instrument Modes**

|  | **Total Cells Scanned** | | | | **Blast Cell Detection Results** | | | |
| --- | --- | --- | --- | --- | --- | --- | --- | --- |
|  |  |  |  |  |  |  |  |  |
| **Case No.** | **XN-9000 WBC Count** | **Cygnus (whole-slide scanning mode)** | **DI-60 (200 mode)** | **Cygnus (200 mode)** | **XN-9000 blood cell analyzer blast alarm** | **Cygnus (whole-slide scanning mode)** | **DI-60 (200 mode)** | **Cygnus (200 mode)** |
|  |  |  |  |  |  |  |  |  |
| **1** | **0.2** | **229** | **103** | **118** | **-** | **5** | **3** | **1** |
| **2** | **0.32** | **191** | **43** | **136** | **+** | **1** | **-** | **-** |
| **3** | **0.4** | **334** | **62** | **200** | **+** | **12** | **1** | **4** |
| **4** | **0.41** | **281** | **34** | **87** | **-** | **5** | **-** | **-** |
| **5** | **0.42** | **121** | **32** | **61** | **+** | **2** | **1** | **-** |
| **6** | **0.73** | **413** | **63** | **141** | **+** | **49** | **4** | **17** |
| **7** | **0.78** | **303** | **44** | **183** | **-** | **2** | **1** | **1** |
| **8** | **1.08** | **556** | **115** | **160** | **+** | **5** | **1** | **-** |
| **9** | **1.17** | **923** | **169** | **199** | **-** | **2** | **-** | **-** |
| **10** | **1.31** | **1136** | **139** | **200** | **+** | **1** | **-** | **-** |
| **11** | **1.63** | **1200** | **44** | **200** | **-** | **1** | **-** | **-** |
| **12** | **1.71** | **1263** | **157** | **157** | **-** | **8** | **-** | **2** |
| **13** | **1.77** | **1428** | **144** | **200** | **-** | **7** | **-** | **1** |
| **14** | **1.78** | **1044** | **24** | **102** | **-** | **6** | **-** | **1** |
| **15** | **1.88** | **1372** | **94** | **200** | **+** | **4** | **-** | **-** |
| **16** | **1.92** | **1560** | **171** | **200** | **+** | **200** | **11** | **9** |
| **17** | **2** | **1676** | **154** | **200** | **-** | **1** | **1** | **1** |
| **P-values** |  | **0.000043** | |  |  |  |  |  |
| **P-values** |  |  | **0.000416** | |  |  |  |  |
| **Overall blast identification rate(%）** | | | | | **47.1%** | **100%** | **52.9%** | **47.1%** |

**(Note: "+" denotes blast cell detection by the instrument; "-" indicates no blast cells were detected)**
